# THE IMPACT OF FOOD INSECURITY ON THE HEALTH AND WELL-BEING OF OLDER ADULTS: THE GROUPS MOST AT RISK

**DOI:** 10.1093/geroni/igz038.2705

**Published:** 2019-11-08

**Authors:** Lauren E Popham, Erin McGovern

**Affiliations:** National Council on Aging, Arlington, Virginia, United States

## Abstract

Older adults who experience food insecurity (4.6 million) often have worse health outcomes. Food insecure older adults consume less nutrients, which puts them at greater risk of developing chronic diseases. They are at increased risk of falls due the impact of poor nutrition on muscle mass, bone density, and balance. Low-income older adults are often forced to choose between buying groceries and paying other bills. The Supplemental Nutrition Assistance Program (SNAP) plays an important role in reducing food insecurity. SNAP enables older adults to buy the nutritious food they need, while freeing up resources to pay for everyday things to meet their health needs such as prescription drugs. Research shows that medication adherence increases when low-income older adults enroll in SNAP. Despite the beneficial impact of enrolling in SNAP, it’s estimated that 55% of eligible adults age 60 and older are not participating in this critical program. To understand which older adults are missing out on SNAP, the National Council on Aging engaged researchers at Leading Age LTSS Center at UMass Boston to analyze data from the 2014 Health and Retirement Study. The results show that some of the most vulnerable older adult populations are less likely to participate in SNAP even though they are eligible (i.e., Hispanic, age 75 and older, those who are not utilizing healthcare, etc.). The findings suggest that more targeted outreach to these groups is needed to ensure that the most vulnerable populations of older adults access this critical benefit.

